# Mass Spectrometry for Diabetic Nephropathy Monitoring: New Effective Tools for Physicians

**DOI:** 10.5402/2012/768159

**Published:** 2012-05-20

**Authors:** Annunziata Lapolla, Simona Porcu, Pietro Traldi

**Affiliations:** ^1^Department of Medicine, University of Padova, Via Giustiniani 2, 35137 Padova, Italy; ^2^Istituto di Scienze e Tecnologie Molecolari, Consiglio Nazionale delle Ricerche, Corso Stati Uniti 4, 35127 Padova, Italy

## Abstract

The main aim of diabetic nephropathy monitoring is to identify molecular markers, that is, to find changes occurring at metabolome and proteome levels indicative of the disease's development. The mass spectrometry methods available today have been successfully applied to this field. This paper provides a short description of the basic aspects of the mass spectrometric methods used for diabetic nephropathy monitoring, reporting and discussing the results obtained using different approaches.

## 1. Introduction

Diabetic nephropathy (DN) is one of the most important chronic complications of diabetes and, because it is associated with an increased frequency of cardiovascular disease, it is an important factor in the morbidity and mortality of the latter [1]. Worldwide, DN is also a major determinant of end-stage renal disease (ESRD) in approximately 40% of patients requiring kidney replacement [1]. The public health burden of DN is consequently very high.

Although a number of factors have been associated with the onset of DN—both genetic [2] and environmental (poor glycemic control, hyperlipidemia, hypertension, smoking) [3]—the pathogenesis of this disease has yet to be thoroughly clarified. On the other hand, a comprehensive understanding of this disease's physiopathology is fundamental to the design of therapeutic approaches capable of preventing the development of DN and of the consequent ESRD [4].

Diabetic nephropathy usually begins with the onset of microalbuminuria, which proceeds to proteinuria, increasing creatininemia and azotemia, and ultimately develops into ESRD. It is worth emphasizing that numerous, potentially reversible functional changes take place in the kidney before proteinuria sets in, including hyperfiltration, hyperperfusion, and an increase in the capillary permeability of the macromolecules. Then come several morphological changes, such as basement membrane thickening, mesangial expansion, glomerulosclerosis, and tubulointerstizial fibrosis; these changes are irreversible and can trigger the onset of ESRD [5, 6]. Measuring urinary albumin excretion is the method generally used to classify DN, and a stable increase in microalbuminuria is considered the first sign of renal damage [7, 8]. About 20–40% of type 2 diabetic patients progress to macroalbuminuria [9], and 40–50% of patients with microalbuminuria also suffer from cardiovascular disease [10]. These data further underscore the importance of identifying patients prone to developing DN and consequently ESRD as early as possible. Although albuminuria is considered one of the main predictors of DN, there is still some controversy concerning its sensitivity and specificity for this purpose [11, 12]. It would, therefore, be useful to identify additional protein markers capable of predicting whether patients with diabetes risk developing DN even many years before the first clinical signs appear, and whether they risk suffering from ESRD; this would certainly reduce the burden and social cost of DN.

## 2. Mass Spectrometric Methods Used to Study Diabetic Nephropathy

The onset of DN is necessarily reflected in a different urinary protein profile. Albuminuria (i.e., the albumin level in urine) is widely used as a diagnostic test for the onset of nephropathy despite persistent doubts as to its efficacy [11, 12]. Developments in new analytical methods focusing on the human proteome may, however, provide physicians with very powerful tools capable of obtaining information on the pathological mechanism(s) behind DN and enabling the efficacy of specific therapies to be assessed.

Mass spectrometry (MS) [13, 14] affords an extremely interesting instrumental approach to describing a patient's urinary profile, and it has been used in various ways in the last decade to map the urinary proteins with a view to identifying the species indicative of the onset of nephropathy.

Given the thousands of proteins and peptides to be found in urine, the method used to identify them must be highly sensitive and specific. A preliminary method used with some success is one- and two-dimensional electrophoresis but, here again, MS is needed to establish the molecular weight and structure of the various proteins separated by the electrophoretic method.

**Figure 1:**
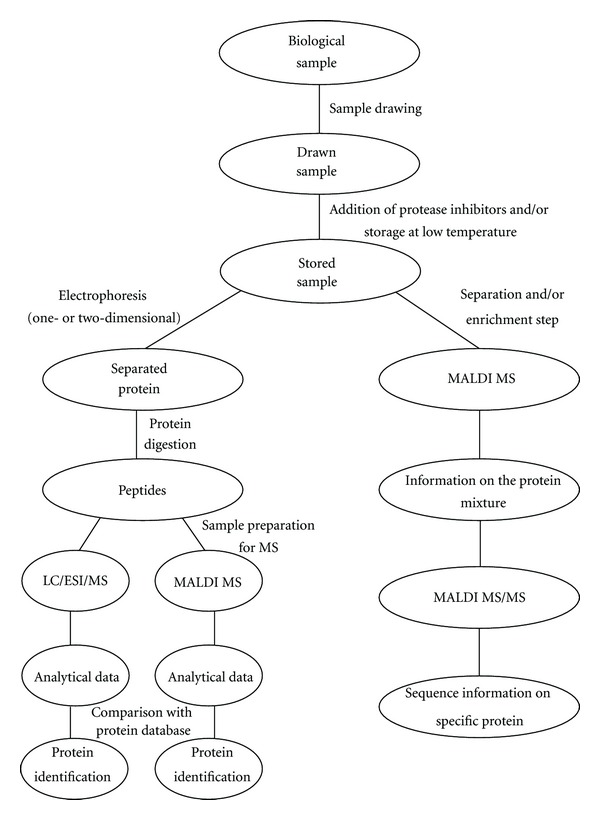
Proposed approaches for proteomic investigations using mass spectrometry.

Generally speaking, two MS approaches have been most widely used to study the urinary proteome (Figure 1) [15]. In one (on the left in Figure 1), the protein mixture is first treated by electrophoresis (be it one- or two-dimensional) to separate the various proteins contained in the mixture. It would be more accurate to define this step as a “partial” separation procedure because neither one- nor two-dimensional electrophoresis have a resolution high enough to separate *all* the different proteins. What is generally seen is more than one protein in a given band (using monodimensional electrophoresis) or spot (in 2D conditions). The denaturing conditions of SDS-PAGE must also be taken into account.

The best results can be obtained with capillary zone electrophoresis (CZE) [15], but this approach is more expensive than 1D or 2D electrophoresis because of the cost of the instrumentation required and the lengthy analysis times.

It is difficult to obtain intact proteins from 1D or 2D electrophoresis gel, due mainly to the small quantities of the analytes. The proteins contained in a band or spot are usually digested enzymatically and the structures of the resulting peptides are then identified using suitable MS procedures. This identification step can be conducted using tandem mass spectrometry (MS/MS) of protonated molecules of the analytes generated by electrospray ionization (ESI) [13]. The quality of the results depends on the mass spectrometric resolution [14]: in high-resolution conditions, structures are assigned on the basis not only of the collisionally induced decomposition products (MS/MS), but also of their elemental composition, obtained from accurate mass measurements. This approach thus provides information on some of the protein's substructures (peptides), not the original protein, and this latter information must be obtained by comparing the tryptic fragments with protein databases to obtain a list of possible proteins with experimentally-determined amino acid sequences, using a parameter to assess the validity of the structural assignment [15].

The two MS methods generally used rely on two different ionization techniques, electrospray (ESI), and matrix-assisted laser desorption ionization (MALDI). The power of these methods has been recognized by the scientific community with the assignment of the 1999 Nobel Prize to John Fenn and Koichi Tanaka, the scientists who developed ESI and MALDI, respectively.

Electrospraying involves spraying the analyte solution generated by a strong electrical field. These conditions prompt the formation of charged droplets of the solution, and evaporation of the solvent leads to an increase in the droplets' surface charge density and to the formation of the protonated analyte ions in the gaseous phase. In the case of proteins, the presence of a large number of basic sites within their structure leads to the formation of multiprotonated ion clusters from which it is easy to calculate the molecular weight of the analyte. The power of ESI is related to the ease with which it can be interfaced on-line with chromatographic (LC) or electrophoretic (CZE) methods, so as to obtain ESI mass spectra for each component separated using the LC or CZE methods.

MALDI is based on the interaction of a laser beam with a solid-state sample consisting of a suitable matrix (usually variously substituted organic aromatic acids) in which the analyte is contained at very low levels (in the typical matrix, the ratio of the analyte is in the order of 10000 : 1). The pulsed laser beam causes ionization of the matrix and a rapid phase change, with the formation of a dense cloud comprising the neutral and ionized matrix and molecules of the analyte. In this region, ion-molecule reactions can occur with a high yield, leading to the formation of protonated molecules of the analyte. MALDI MS gives rise to the formation of singly protonated species, so it can be useful for characterizing protein mixtures without any need for chromatographic separations. In other words, from a simple MALDI spectrum we can obtain a “protein profile” that correlates well with the proteins most abundant in the mixture. The drawback of the MALDI method is its low “dynamic range” (in the order of 100–1000), which means that MALDI only allows for a linear response between quantity and signal to be obtained within this range. There may also be ion suppression phenomena, which can give rise to quantitative data that are not always reliable. To give an example, introducing hemoglobin makes the two signals related to globins *α* and *β* detectable with a high intensity, but their abundance ratio is not 1 : 1, as we might expect, but 0.8 : 1, indicating that *β* globin is ionized in a higher yield [16].

To obtain more specific protein profiles, samples can be deposited on different surfaces prior to laser irradiation. This approach is usually called SELDI (surface-enhanced laser desorption/ionization), and it involves depositing the sample and matrix on a surface modified to have a specific chemical functionality before proceeding with laser ionization. Surfaces commonly used in this context include weakly positive ion exchange, hydrophobic surfaces (similar to those used in C_6_–C_12_ reverse phase chromatography), metal-binding surfaces, and strong anion exchangers. Surfaces can also be functionalized with antibodies, other proteins, or DNA. Only the analytes with a good affinity with a given treated surface will remain fixed on the surface, while all the other compounds are removed by washing. Then SELDI can be used to enrich the substances of interest.

As shown in the flow chart in Figure 1, using chromatographic (LC) or electrophoretic (1D or 2D gel electrophoresis, or CZE) leads to the separation of the components in a complex biological substrate and, when connected on-line with MS (operating in ESI conditions) or off-line with a MALDI/MS source (using suitable robotic devices), these methods enable us to establish at least the molecular weight of the different proteins/peptides.

We say “at least” because the protonated molecular species can be used in collisional experiments: they are selected and decomposed by making them collide with a target gas. Their collision prompts an internal energy deposition, which promotes their fragmentation and provides valid information on the protein/peptide amino-acid sequence.

As described below, interesting results in the field of urinary proteins have been obtained with all these instrumental approaches, but the most powerful is CZE/MS.

Capillary electrophoresis can be used to separate ion species contained in a solution based on their charge status, hydrodynamic radius, and functional form. In other words, electrically charged analytes move in a conducting medium due to the action of an electrical field. Many different detectors can be used to identify the different analytes emerging from the capillary tube, and mass spectrometry (usually operating in ESI conditions) is the most sensitive and specific. By comparison with LC, in which the molecular species are separated by means of their affinity for the stationary phase, CZE shows a remarkably better resolution, capable of separating the single components contained in a highly complex natural matrix.

The results obtained by the different approaches briefly outlined above, in terms of characterizing urinary protein profiles, are described in more detail in Sections 3–6.

## 3. Results Obtained by Surface-Enhanced Laser Desorption/Ionization

SELDI/MS generally produces valid results for protein profiling in complex natural matrices. It has been and still is used to identify any changes in the urinary protein profiles of patients with diabetes and other diseases.

Suitable surfaces are used to select and concentrate the analytes of interest.

The weakness of the method lies in the quality of MALDI spectra, which is not as good as the quality achievable using more complex MALDI machines. Any structure assigned by means of MS/MS experiments cannot be obtained by SELDI; the only analytical information it provides is the molecular weight of the different proteins/peptides in the sample.

In one study, SELDI was used to develop a method for predicting diabetic nephropathy [17]: urine samples from 31 type 2 diabetic patients were analyzed systematically over a decade, correlating changes in their urinary protein profiles with any onset of DN. The SELDI data were considered as a fingerprint of an individual's physiological/pathological status, and differences were observed from a morphological standpoint, without considering the possible protein structures. This method, nonetheless, showed that urinary proteomic profiling with SELDI can identify normoalbuminuric subjects with type 2 diabetes who will subsequently develop DN.

SELDI mass spectrometry (using SAX2 protein assays) was used to compare urinary protein profiles in four different groups of subjects, that is, type 2 diabetics (DM; *n* = 45) with no nephropathy or microalbuminuria (DM WNP), patients with DM and macro- or microalbuminuria (DM-NP; *n* = 38), patients with proteinuria due to nondiabetic renal disease (*n* = 34), and healthy controls (*n* = 45). Strongly discriminating proteins were identified and isolated using anion exchange, reversed-phase fractionation, gel electrophoresis, and MS. One protein detected at *m/z* 6188 was released in great abundance in the urine of healthy controls and nephropathic cases without diabetes, and in cases of DM WNP, but not in DM-NP patients [18]. Other proteins were detected at *m/z* 14766 (selectively excreted in the urine of DM-NP patients) and *m/z* 11774 (significantly excreted by patients with proteinuria and DM-NP). These last two proteins were structurally identified as *β*_2_-microglobulin and UbA52 (a ubiquitin ribosomal fusion protein), respectively. Both could be considered interesting diagnostic markers. In the kidney, UbA52 occurs exclusively in the renal tubules and its expression was found proportional to the individual's blood glucose concentrations. Its expression is also regulated by oxidative and carbonyl stress, which are important factors in the pathophysiology of DN and apoptosis. Quantifying the ubiquitin degradation product detected at *m/z* 6188 (the level of which declined from healthy to DM-NP patients) could provide interesting information on the development of DN.

## 4. Result Obtained by Liquid Chromatography and Mass Spectrometry

The combination of liquid chromatography and mass spectrometry (LC/MS) has been used mainly to analyze digested urinary proteins separated by 1D and 2D gel electrophoresis. In this frame, Rao et al. recently conducted an extensive study, taking the proteomic approach, to identify possible biomarkers of DN [19]. They used DIGE (*Differential in Gel Electrophoresis)* followed by tryptic peptide analysis with LC/MS/MS to identify seven proteins that were upregulated and four that were downregulated with increasing albuminuria levels.

This was achieved on the basis of amino acid sequences obtained by MS/MS experiments performed on the tryptic digestion products, and the resulting data were used for a Lynx Global Server search. *De novo* sequencing was done with a PEAKS algorithm combined with the Open Sea alignment algorithm. It is worth emphasizing that this approach led to the identification of 62 specific proteins belonging to several functional groups (e.g., cell development, cell organization, defensive response, metabolism, and signal transduction).

The proteins downregulated in DN were identified as transthyretin, apolipoprotein A–I, *α*_1_-microglobulin/bikunin precursor, and plasma retinol-binding protein.

Seven proteins were upregulated (>1.5-fold; *P* < 0.05) in cases of DN with macroalbuminuria by comparison with diabetics without albuminuria. Normalized volumes of these upregulated proteins also increased gradually across three categories of diabetic patients with normal, micro- and macroalbuminuria, indicating the proteins' positive association with the progression of DN. The *α*_1B_-glycoprotein rose the most overall (with a 7.0-fold increase), followed by zinc-*α*_2_-glycoprotein (5.9-fold), *α*_2_-HS-glycoprotein (4.7-fold), vitamin D-binding protein (VDBP) (4.8-fold), calgranulin B (3.9-fold), *α*_1_-antitrypsin (A1AT) (2.9-fold), and hemopexin (2.4-fold).

Compared with healthy controls, VDBP exhibited the greatest increase (11.1-fold) followed by zinc-*α*_2_-glycoprotein (6.0-fold), *α*_2_-HS-glycoprotein precursor (2.3-fold), and A1AT (2.2-fold).

Identifying the metabolic changes in these proteins can be seen as a good starting point for elucidating the mechanisms behind the pathogenesis of DN.

More recently, Riaz et al. [20] completed an in-depth study using a very powerful separation method, that is, 2D liquid chromatography with chromatofocusing in the first dimension and reversed-phase chromatography in the second, followed by MS analysis. This method was first used to separate the urinary proteins and ascertain their molecular weight by MALDI/MS. To obtain structural information, the proteins were digested and the peptides obtained were analyzed by LC/MS and LC/MS/MS. The resulting data were then compared with the human subset in the Swiss-Prot protein database, and the protein assignments based on these data were compared with the molecular weights of the proteins obtained by MALDI.

The Riaz et al. investigation proved very highly specific thanks to the high-resolution chromatographic method adopted, the analysis of the molecular weight of intact urinary proteins, the identification of the proteins based on protein digestion, and the structural assignment of the digestion peptides based on the search in the database.

The transthyretin, *α*_1_-microglobulin/bikunin precursor, and haptoglobin precursor levels were 30.8%, 55.2%, and 81.45% lower in the diabetics than in the controls, while the levels of albumin, zinc-*α*_2_-glycoprotein, retinol binding protein 4, and E-cadherin were 486.5%, 29.23%, 100%, and 693% higher, respectively. Changes in the levels of these protein biomarkers have been reported in other pathological states and assessing their levels will be helpful in the early diagnosis and prognosis of diabetes mellitus type 2. These data were interpreted on the assumption that bikunin is an important anti-inflammatory substance and a decline in its levels in pathological conditions may influence anti-inflammatory response. Lower levels of urinary *α*_1_-microglobulin indicate a proximal tubular dysfunction and could serve as an adjunctive biomarker (in addition to albuminuria levels) for the early detection of nephropathy in diabetic subjects.

Among the different LC/MS approaches used to study the urinary proteome, we must also mention the study by Tyan et al. [21], based on nano-LC/MS/MS after enzymatic digestion. The instrumental setup enabled the identification of 2,283 peptides, corresponding to 311 proteins. The method was unfortunately developed on urine from healthy subjects, but its high specificity and the small quantity of sample needed for the analysis suggest that this method could be extremely effective for studying changes in urinary protein profiles in disease.

## 5. Results Obtained by Capillary Zone Electrophoresis and Mass Spectrometry

Meanwhile, an extensive investigation was conducted by Mischak et al. [22], focusing mainly on assessing diabetes-related renal damage in humans in terms of urinary protein profile. Using a highly-specific method based on CZE coupled with ESI-MS, the authors identified a “normal” urinary polypeptide pattern in 39 healthy subjects that clearly differed from the pattern seen in 112 patients with type 2 DM. This prompted the identification of a specific “diabetic” pattern of polypeptide excretion. This approach led to the detection of peptides pointing to diabetic renal damage in patients with high albuminuria levels.

The same instrumental approach (CZE/ESI/MS) was used to identify urinary protein patterns in type 1 diabetic adolescents with early diabetic nephropathy [23]. Among more than 1,000 different polypeptides (in the mass range of 800-66,500 Da), the method was able to reveal a specific cluster of 54 polypeptides found only in the urine of diabetic patients.

In another study, CZE/MS analysis was used to analyze changes in urinary polypeptide patterns during treatment with the angiotensin II receptor blocker (ARB) candesartan [24]: the treatment was found to significantly change 15 of the 113 polypeptides characteristic of macroalbuminuric patients, suggesting that this analytical approach can be useful for monitoring the efficacy of pharmacological treatments.

CZE coupled with ESI-MS was also used recently by Rossing et al. [25] to elucidate the panorama of urinary proteomics in diabetes and chronic kidney disease (CKD). Studying 305 subjects identified a panel of 40 biomarkers capable of distinguishing diabetic patients from healthy subjects with a 89% sensitivity and 91% specificity. The distinction between cases of DN and other CKDs reached a sensitivity of 81% and a specificity of 91%. Many of the biomarkers identified were fragments of collagen type 1 and they were in clearly lower quantities in patients with diabetes or DN. The uromodulin fragment 589–607 was also detected.

Both the classic proteomics approach and CZE-based measurements are highly-specific methods, but they have the drawback of being difficult to use in clinical chemistry laboratories. They both take time and demand experienced personnel and expensive equipments. Alternative analytical approaches would, therefore, certainly be of interest.

The power of CZE-MS in the discovery, validation, and clinical application of biomarker was exhaustively reviewed very recently by Mischak and Schanstra [26], who describe the instrumental setup in detail and draw a thorough comparison between the CZE-MS and LC-MS approaches. The authors report that the main advantages of CZE are its robustness, short run times and rapid reconditioning, which are helpful when large numbers of heterogeneous samples containing interfering compounds are being analyzed. The main disadvantage of CZE lies in its limited loading capacity, which negatively reflects on the feasibility of performing MS/MS experiments. CZE fractions can be collected and spotted off-line onto a MALDI target plate, however. The polypeptides of interest can be sequenced by means of MALDI TOF/TOF experiments, though the authors emphasize that this approach is often unsuccessful, probably due mainly to its low sensitivity and insufficient mass accuracy.

Considering the great complexity of the urinary protein profile, as described in detail by CZE/MS, the subsequent data analysis and statistics are an important issue when it comes to identifying potential markers of disease. In the case of CZE/MS, the migration time does not change very much and an internal standard can be used to calibrate the migration time successfully. These processes then lead to the assignment of unique, readily reproducible parameters that identify each peptide mass and migration time; signal amplitude can be considered a valid measure of relative abundance. These results can be used for statistical analyses, then the data sets can be used to conduct comparative studies by multivariate analysis.

A recent paper [27] confirmed the strength of this approach: a multicenter validation of urinary proteomic biomarkers specific for DN was implemented blindly using CZE-MS, and the resulting data were assessed using the model previously developed for DN. Samples from 148 Caucasian type 2 diabetic patients exhibiting albuminuria >300 mg/dL (cases), recruited at three different European centers, were analyzed and compared with those of 82 diabetic patients matched for gender and diabetes duration (controls). Sixty previously identified peptides differed significantly between controls and patients. All the data processing was based on the classification previously developed and validated by Rossing et al. [25] for differentiating between type 1 diabetic patients with and without macroalbuminuria after CZE/MS analysis, obtaining a 100% sensitivity and a 97% specificity.

It is worth noting that it was only in under 10% of cases and controls that the CZE/MS classification did not match the clinical data, but further evaluation of the patients revealed a progression to DN in some of the false positive, originally classified as DN controls patients. This point is of interest, indicating that the proposed method is effective in identifying the early stages of DN.

Given its high specificity and the consequently large number of urinary peptides identified, the CZE/MS approach provides to a broad panorama of the urinary proteome that can be used successfully in biochemical considerations strictly relating to pathophysiology [28]. In particular, many urinary collagen fragments have been identified and their levels are significantly altered in diabetes. Monitoring these collagen fragments enables type 1 and type 2 diabetic patients to be distinguished from one another: specific collagen fragments are associated with diabetes, and with the type of diabetes, pointing to changes in collagen turnover and extracellular matrix as a hallmark of the molecular pathophysiology of diabetes. The data obtained by CZE-MS show that the abundance of collagen fragments (collagen alpha-1 [I] and [III]) decreases in the passage from being healthy to acquiring diabetes and this drop is more evident in cases of type 2 diabetes with no evidence of chronic kidney disease than in type 1. Type 2 diabetic patients also exhibited lower levels of microalbuminuria and higher levels of GFR than in type 1 DM patients.

These findings may indicate that the impaired collagen degradation mechanisms differ (or occur with different yields) in the two types of diabetes.

## 6. Results Obtained by Matrix-Assisted Laser Desorption/Ionization Mass Spectrometry

MALDI is a very different analytical method from those described so far. Two different approaches are generally used to apply MALDI in the proteome field. One is based on analyzing the enzymatic digestion products of previously separated proteins/peptides (usually by 2D gel electrophoresis). This method quickly provides the fingerprint of the digestion products to use in library searches. If a higher specificity is needed, MS/MS experiments can be performed on the digestion products to obtain their amino acid sequences.

The second approach is based on the MALDI analysis of intact urinary extracts, without any sample pre treatment. Alongside the use of cutoff membranes to exclude the higher-molecular-weight proteins (e.g., albumin), using different matrices can lead to quite a complete fingerprint of the urinary protein profile.

In both cases, a comparison must be drawn between healthy subjects and patients with different degrees of DN to identify possible markers of the disease and thereby clarify the metabolic changes responsible for it.

As mentioned in Introduction, the MALDI method does not need coupling with chromatographic or electrophoretic systems. The protein/peptide mixtures are analyzed directly, generating a “fingerprint” of the mixtures. A weakness of the method lies in its limited dynamic range, which—together with possible suppression effects—may produce only a partial view. The method's speed of analysis, very wide mass range (up to 500,000 Da), and sensitivity (at fentomol level), nonetheless, make MALDI a powerful tool for proteomic studies.

The investigation conducted by Jiang et al. [29] is a good example of the first approach. Urine samples from type 2 diabetic patients with normal albuminuria (DM), microalbuminuria (DN1), or macroalbuminuria (DN2) were analyzed by comparison with those from a control group. The first step of this study relied on fluorescence-based difference gel electrophoresis (DIGE) followed by MS to identify possible novel biomarkers. A total of 12 differently expressed proteins came to light, 8 of them significantly upregulated and 4 significantly downregulated in the DN groups versus the control group. Then the researchers focused on a novel protein, E-cadherin, because of the magnitude of its change (it was found upregulated 1.3-fold, 5.2-fold, 8.5-fold in the D, DN1 and DN2 groups versus controls). This protein was further studied in the urine by western blot and in renal biopsies using immunohistochemical methods. Its urinary levels were also obtained by ELISA.

E-cadherin is a 120 kDa transmembrane glycoprotein that plays a critical part in calcium-dependent cell-to-cell junctional adherence. It is mainly located in the tubular epithelial cells, with a key role in maintaining their structural integrity. Renal ischemia and apoptosis could cause E-cadherin degradation and cleavage via proteolytic enzyme activation, leading to an increase in its levels in the urine. Immunohistochemical data show a corresponding decrease in E-cadherin expression.

The lower expression of E-cadherin in the kidney may, therefore, correlate with the increased urinary levels of E-cadherin seen in DN patients, but further studies are needed to better clarify the role of this marker in the pathogenesis of DN.

MALDI/MS was used by Kumar et al. [30] to investigate the proteomics of renal diseases. The approach taken in this case was based on analyzing urine samples from patients with different renal conditions (end-stage renal disease, nephropathic syndrome, and microalbuminuria) using 2D electrophoresis, in gel proteolytic digestion, and MALDI analysis of the digestion products. Patients with kidney failure had more low-molecular-weight proteins, while those with nephrotic syndrome had a greater abundance of high-molecular-weight proteins.

For patients with microalbuminuria, the proteins considered the most representative were Zn-*α*-2-glycoprotein, alpha-1-microglobulin, and *α*-1-acid glycoprotein, *α*-1-acid glycoprotein 2.

On the whole, the protein profiles obtained in different clinical conditions were quite different, pointing to a possible diagnostic use of this method.

**Figure 2:**
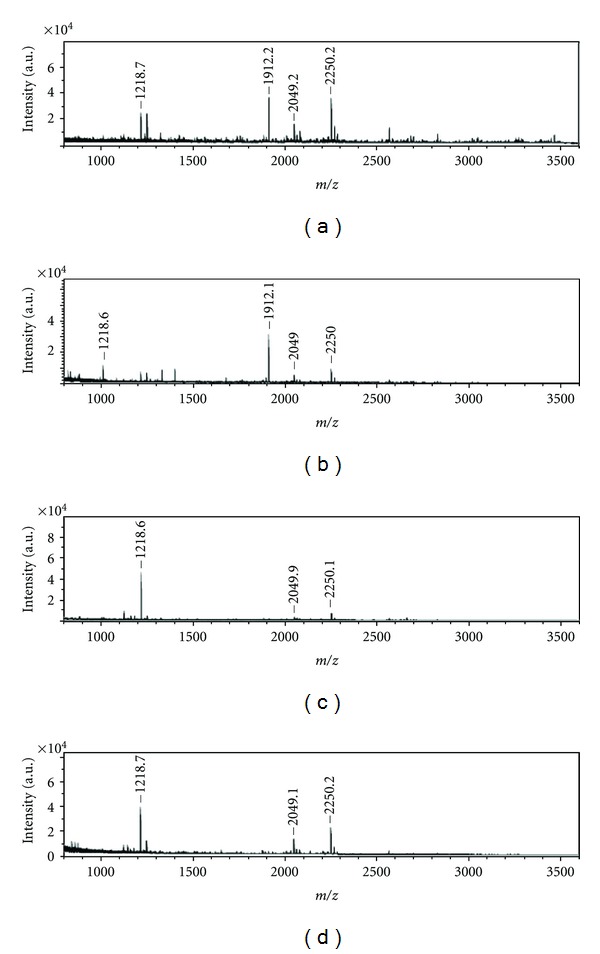
MALDI mass spectra of urine samples from: (a) control subject, (b) diabetic patient, (c) nephropathic patient, and (d) diabetic-nephropathic patient [31].

Unlike the approaches described before, MALDI has been used for the direct fingerprinting of urinary proteins. The results of a preliminary study [31] on urine samples from type 2 diabetic patients, patients with renal disease, type 2 diabetic patients with renal disease, and healthy controls showed that the MALDI spectra of the low-molecular-weight fraction of the peptides in urine samples varied considerably within each group, mainly as concerned their relative abundance. This variability would suggest that the method is unsuitable for characterizing groups in different pathological states. Some differences became clearly apparent, however, on comparing the spectra belonging to the different groups (see Figure 2, e.g.). It was immediately obvious that the spectra from diabetic patients and healthy subjects were quite similar: in both cases, the most abundant peak was at *m/z* 1912, while other, less abundant peaks were seen in both cases at *m/z* 1219 and 2049 (this last species was underexpressed in samples from diabetic patients). The peaks characteristic of the samples from healthy subjects and diabetics were much lower in the case of nephropathic and diabetic-nephropathic patients (whose most abundant peak was at *m/z* 1219), whereas the ion at *m/z* 1912 was strongly suppressed in these nephropathic patients.

The MS/MS spectrum of the ion at *m/z* 2049, obtained by means of TOF-TOF experiments, was identical for all the samples examined and the search using the Protein-Pilot v.2.1 software in the Uniprot Database indicated that its sequence is NGDDGEAGKPGRHypGERGPHypGP, corresponding to the precursor of the collagen *α*-1 (I) chain [32]. Using the same approach, the ion at *m/z* 1912 was found to have the sequence SGSVIDQSRVLNLGPITR and to coincide with the uromodulin precursor. Finally, the ion at *m/z* 1219 was found to coincide with the IGPHypGPHypGLMGPP sequence of the precursor of the collagen *α*-5 (IV) chain.

**Figure 3:**
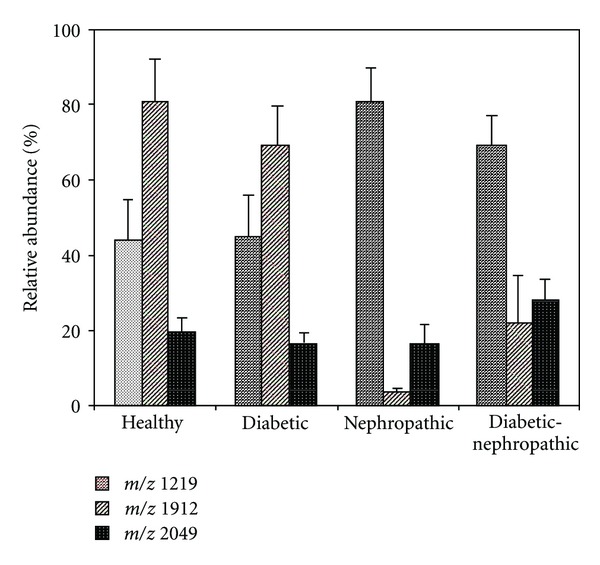
Histograms of the abundance of ions at *m/z* 1219, 1912, and 2049 found in urine samples of the subjects under study. Data are expressed as mean ± SEM [31].

The histograms of the abundance of the ions at *m/z* 1219, 1912, and 2049 in urine samples from the subjects under study are shown in Figure 3.

The underexpression of the uromodulin fragment seen in nephropathic patients with advanced renal disease and in diabetic patients with advanced nephropathy might relate to an alteration of the apical cell membrane of the thick ascending limb (TAL) epithelial cells, while the collagen fragments might be explained by an alteration of the renal basal glomerular membrane.

**Figure 4:**
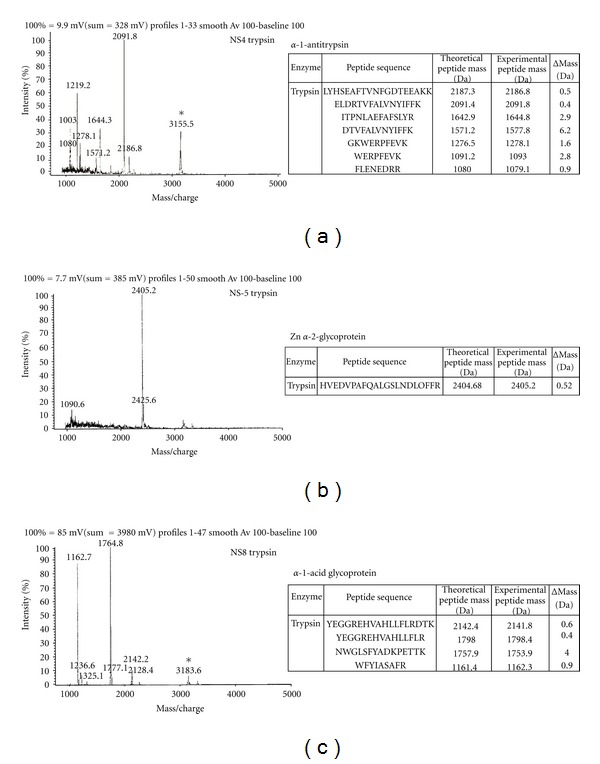
MALDI-TOF analysis of urinary-digested 2D electrophoretic protein spots from nephrotic syndrome patients (protein spots were visualized by Coomassie staining and labelled NS1 to NS8). (a) Protein spots, corresponding to NS4, were subjected to in-gel digestion with trypsin. On the left panel, tryptic peptide masses were obtained by MALDI-TOF analysis; on the right panel, comparison of experimentally determined peptide masses of NS4 with theoretical peptide masses of *α*-1-antitrypsin. (b) MALDI-TOF profiles of tryptic peptides from protein spots corresponding to NS5 (Left panel) were obtained by MALDI-TOF analysis: comparison of experimentally determined peptide masses of RF2 with theoretical peptide masses of Zn-*α*-2-glycoprotein (Right panel). (c) MALDI-TOF profiles of tryptic peptides from protein spots corresponding to NS8 (Left panel) were obtained by MALDI-TOF analysis: comparison of experimentally determined peptide masses of NS8 with theoretical peptide masses of *α*-1-acid glycoprotein 2 (Right panel). Background peaks are marked with ‘∗' in the MALDI-TOF spectra [30].

Further investigations on a larger population confirmed these findings [33]. The results obtained underwent statistical analysis, and the *P* values for the differences observed indicate that they are statistically significant when comparison are drawn between all patients and healthy controls, between diabetics with normal or microalbuminuria and nephropathic cases with advanced renal disease, and between diabetics with normal or microalbuminuria and diabetics with advanced nephropathy. The scatter plot shows the strict inverse relationship between the abundance of ions at *m/z* 1912 and 1219, with the correlation coefficient being particularly high (*r* = 0.921, *P* < 0.001). The relationship between the true positive rate (sensitivity) and the false positive rate (1-specificity) for every possible cut-off in the abundance of the ion species considered was investigated using the receiver-operating characteristic (ROC) curve. The data obtained indicate that a clear distinction can easily be drawn between nephropathic patients with advanced renal disease or diabetics with advanced nephropathy and healthy controls using this approach (Figure 4). 

## 7. Future Trends to Be Expected

The development of new analytical methods based on mass spectrometry has been generating interesting and valid results on the changes induced by diabetes in an individual's urinary protein profile.

Two different approaches have been taken, both based on a first separation step followed by MS analysis; these two steps can be conducted on-line (as in the case of LC or CZE/MS) or off-line (as in the case of 1D and 2D electrophoresis). The protein is then generally characterized by enzymatic digestion of the separated species and MS is used to ascertain the molecular weight of the digestion products.

When analyzed with the aid of protein data banks, the results enable the protein responsible for the production of the digestion products to be identified. In the case of CZE/MS, the enzymatic digestion phase is unnecessary and the intact proteins are characterized on the strength of their migration time and molecular weight. Finally, urinary protein maps can be obtained by MALDI without pretreating the sample.

To give an overview of the high number of data obtained by the MS approaches, the diabetes urinary protein biomarkers identified by the investigations above described are reported in Table 1.

In this table, the peptide/proteins are listed for increasing *m/z* values. For each of them, the name, the biological processes which are involved, the type of disease, the regulation together with the analytical method employed for their detection, and the related reference are given.

In a further, wider table (available as supplementary material available at doi:10.5402/2012/768159) all the published data for both attributed and unattributed proteins, are listed.

All these approaches have given us a clear idea of the changes occurring in the urinary protein profile due to the development of diabetes. Some markers obtained in this way have already been validated and these new analytical approaches can be expected to be applied at clinical laboratory level in the near future, giving physicians new diagnostic tools for investigating the clinical status of diabetic subjects and consequently developing suitable therapeutic approaches.

It is worth emphasizing that such new mass spectrometry approaches are constantly evolving with the introduction of instrumentation assuring an ultra-high specificity and sensitivity. But for the time being, these instrumental approaches demand highly experienced scientists and plenty of funds for purchasing the necessary equipment—two aspects that currently make these systems unsuitable for routine clinical chemistry measurements.

## Supplementary Material

Diabetes urinary protein biomarkers. Accession number in National Center for Biotechnology Information databases. DM: Diabetic patients; DN: Diabetic nephropathy; DM-NP: Diabetic patients with macro- or microalbuminuria.

## Figures and Tables

**Table 1:** Diabetes urinary protein biomarkers. ^a^Accession number in National Center for Biotechnology Information databases. DM: Diabetic patients; DN: Diabetic nephropathy; DM-NP: Diabetic patients with macro- or microalbuminuria.

45.861	Haptoglobin precursor	P00738	Metabolism	Type 2 DM-NP	Downregulated	DIGE followed MALD1-TOF-MS	[29]
46,707	*α*1-antitrypsin	P01009	Defense response	Type 2 DM-NP	Upregulated	DIGE followed LC/MS/MS peptide analysis	[19]
51,643	*α*1-microglobulin/bikunin precursor	P02790	Transport	Type 2 DM-NP	Downregulated	DIGE followed LC/MS/MS peptide analysis	[19]
51,643	Hemopexin	P02790	Defense response	Type 2 DM-NP	Upregulated	DIGE followed LC/MS/MS peptide analysis	[19]
52,964	Vitamin D-binding protein	P02774	Transport	Type 2 DM-NP	Upregulated	DIGE followed LC/MS/MS peptide analysis	[19]
54,239	*α*1B- Glycoprotein	P04217	Function not assigned	Type 2 DM-NP	Upregulated	DIGE followed LC/MS/MS peptide analysis	[19]
71,317	Albumin	P02768	Transport	Type 2 DM	Upregulated	Two-Dimensional Liquid Chromatography followed by MALDI	[20]
71,317	Serum albumin precursor	P02768	Transport	Type 2 DM-NP	Upregulated	DIGE followed MALDI-TOF-MS	[29]
72,451	Uromodulin precursor	P07911	Defense responsc	Type 2 DM-NP	Downregulated	DIGE followed MALDI-TOF-MS	[29]
72,984	Kininogen precursor	P01042	Defense response	Type 2 OM-NP	Upregulated	DIGE followed MALDI-TOF-MS	[29]
97.853	Epithelial-cadherin	P12830	Cell adhesion	Type 2 DM	Upregulated	Two-Dimensional Liquid Chromatography followed by MALDI	[20]
97,853	Epithelial-cadherin	P12830	Cell adhesion	Type 2 DM-NP	Upregulated	DIGE followed MALDI-TOF-MS	[29]
2,049	Collagen *α*-1 (I) chain precursor	P02452	Structural Component	Type 2 DM-NP and NP	Upregulated	MALDl/TOF/TOF	[31]
2,063	Collagen alpha-1 (III) chain	P02461	Structural Component	Type 2 DM	Downregulated with respect to type i dm	CZE-MS	[28]
2,192	Collagen *α*-1(I) chain	P02452	Structural Component	Type 2 DM-NP	Downregulated	CZE coupled with ES1 mass spectrometry	[25]
2,192	Collagen alpha-1(I) chain	P02452	Structural Component	Type 2 DM	Downregulated with respect to type 1dm	CZE-MS	[28]
2,339	Collagen alpha-1(I) chain	P02452	Structural Component	Type 2 DM	Downregulated with respect to type 1 dm	CZE-MS	[28]
2,377	Collagen *α*-1(I) chain [227 to 250]	P02452	Structural Component	Type 2 DM-NP	Downregulated	CZE coupled with ESI mass spectrometry	[25]
2,430	Collagen alpha-1(I) chain	P02452	Structural Component	Type 2 DM	Downregulated with respect to type 1 dm	CZE-MS	[28]
2,487	Collagen alpha-1(I) chain	P02452	Structural Component	Type 2 DM	Downregulated with respect to type 1dm	CZE-MS	[28]
2,687	Collagen alpha-1(I) chain	P02452	Structural Component	Type 2 DM	Downregulated with respect to type 1 dm	CZE-MS	[28]
3,092	Collagen alpha-1(I) chain	P02452	Structural Component	Type 2 DM	Downregulated with respect to type 1 dm	CZE-MS	[28]
3,617	Collagen alpha-2(I) chain	P08123	Structural Component	Type 2 DM	Downregulated with respect to type i dm	CZE-MS	[28]
3,802	Collagen alpha-2(I) chain	P08123	Structural Component	Type 2 DM	Downregulated with respect to type 1 dm	CZE-MS	[28]
11,773	Ig Kappa Chain C region	P01834	Defense response	Type 2 DM-NP	Upregulated	DIGE followed MALDI-TOF-MS	[29]
11,774	*β*2-microglobulin	P61769	Defense response	Type 2 DM-NP with Proteinuria	Upregulated	anion exchange, reversed-phase fractionation, gel electrophoresis and SELDI-TOF MS	[18]
13,234	Calgranulin B	P06702	Defense response	Type 2 DM-NP	Upregulated	Using DIGE followed LC/MS/MS peptide analysis	[19]
2,049	Collagen *α*-1 (I) chain precursor	P02452	Structural Component	Type 2 DM-NP and NP	Upregulated	MALD/TOF/TOF	[31]
2,063	Collagen alpha-1 (III) chain	P02461	Structural Component	Type 2 DM	Downregulated with respect to type 1 dm	CZE-MS	[28]
2,192	Collagen *α*-1(I) chain	P02452	Structural Component	Type 2 DM-NP	Downregulated	CZE coupled with ESI mass spectrometry	[25]
2,192	Collagen alpha-1 (I) chain	P02452	Structural Component	Type 2 DM	Downregulated with respect to type 1 dm	CZE-MS	[28]
2,339	Collagen alpha-1 (I) chain	P02452	Structural Component	Type 2 DM	Downregulated with respect to type 1 dm	CZE-MS	[28]
2,377	Collagen *α*-1 (I) chain [227 to 250]	P02452	Structural Conioonent	Type 2 DM-NP	Downregulated	CZE coupled with ESI mass spectrometry	[25]
2,430	Collagen alpha-1 (I) chain	P02452	Structural Component	Type 2 DM	Downregulated with respect to type 1 dm	CZE-MS	[28]
2,487	Collagen alpha-1 (I) chain	P02452	Structural Component	Type 2 DM	Downregulated with respect to type 1 dm	CZE-MS	[28]
2,687	Collagen alpha-1 (I) chain	P02452	Structural Component	Type 2 DM	Downregulated with respect to type 1 dm	CZE-MS	[28]
3,092	Collagen alpha-1 (I) chain	P02452	Structural Component	Type 2 DM	Downregulated with respect to type 1 dm	CZE-MS	[28]
3,617	Collagen alpha-2(I) chain	P08123	Structural Component	Type 2 DM	Downregulated with respect to type 1 dm	CZE-MS	[28]
3,802	Collagen alpha-2(l) chain	P08123	Structural Component	Type 2 DM	Downregulated with respect to type 1 dm	CZE-MS	[28]
11,773	Ig Kappa Chain C region	P01834	Defense response	Type 2 DM-NP	Upregulated	DIGE followed MALDI-TOF-MS	[29]
11,774	*β*2-microglobulin	P61769	Defense response	Type 2 DM- NP with Proteinuria	Upregulated	anion exchange, reversed-phase fractionation, gel electrophoresis and SELDI-TOF MS	[18]
13,234	Calgranulin B	P06702	Defense response	Type 2 DM-NP	Upregulated	Using DIGE followed LC/MS/MS peptide analysis	[19]
45,861	Haptoglobin precursor	P00738	Metabolism	Type 2 DM-NP	Downregulated	DIGE followed MALDI-TOF-MS	[29]
46,707	*α*1-antitrypsin	P01009	Defense response	Type 2 DM-NP	Upregulated	DIGE followed LC/MS/MS peptide analysis	[19]
51,643	*α*1-microglobulin/bikunin precursor	P02790	Transport	Type 2 DM-NP	Downregulated	DIGE followed LC/MS/MS peptide analysis	[19]
51,643	Hemopexin	P02790	Defense response	Type 2 DM-NP	Upregulated	DIGE followed LC/MS/MS peptide analysis	[19]
52,964	Vitamin D-binding protein	P02774	Transport	Type 2 DM-NP	Upregulated	DIGE followed LC/MS/MS peptide analysis	[19]
54,239	*α*1B-Glycoprotein	P04217	Function not assigned	Type 2 DM-NP	Upregulated	DIGE followed LC/MS/MS peptide analysis	[19]
71,317	Albumin	P02768	Transport	Type 2 DM	Upregulated	Two-Dimensional Liquid Chromatography followed by MALDI	[20]
71,317	Serum albumin precursor	P02768	Transport	Type 2 DM-NP	Upregulated	DIGE followed MALDI-TOF-MS	[29]
72,451	Uromodulin precursor	P07911	Defense response	Type 2 DM-NP	Downregulated	DIGE followed MALDI-TOF-MS	[29]
72,984	Kininogen precursor	P01042	Defense response	Type 2 DM-NP	Upregulated	DIGE followed MALDI-TOF-MS	[29]
97,853	Epithelial-cadherin	P12830	Cell adhesion	Type 2 DM	Upregulated	Two-Dimensional Liquid Chromatography followed by MALDI	[20]
97,853	Epithelial-cadherin	P12830	Cell adhesion	Type 2 DM-NP	Upregulated	DIGE followed MALDI-TOF-MS	[29]
