# Supplementary material for: Mass Spectrometry for Diabetic Nephropathy Monitoring: New Effective Tools for Physicians
Source: ISRN Endocrinol. 2012 May 20;2012:768159. doi: 10.5402/2012/768159 (PMC3363283; doi:10.5402/2012/768159)
Supplement: Supplementary file 1 — Diabetes urinary protein biomarkers. Accession number in National Center for Biotechnology Information databases. DM: Diabetic patients; DN: Diabetic nephropathy; DM-NP: Diabetic patients with macro- or microalbuminuria. [file 768159.f1.pdf]

Supplementary table. Diabetes urinary biomarkers: attributed and unattributed proteins. [a]Accession number in National Center for Biotechnology Information databases.
DM= Diabetic patients; DN= Diabetic nephropathy; DM-NP= Diabetic patients with macro- or microalbuminuria.

m/z	PROTEIN NAME	ACCESSION NUMBER[a]	BIOLOGICAL PROCESS	DISEASE	REGULATION	METHOD/S	REFERENCE
482	Alpha-2-HS-glycoprotein [321-339]	P02765	Mineral balance	Type 2 DM	up-regulated	CZE followed by Orbitrap (ETD)	*Alkhalaf A et al. PLoS One. 2010;5(10):e13421*
544	Beta-2-microglobulin [59-81]	P61769	Defense response	Type 2 DM	up-regulated	CZE followed by Orbitrap (ETD)	*Alkhalaf A et al. PLoS One. 2010;5(10):e13421*
605	CD99 antigen [97-129]	P14209	Cell adhesion	Type 2 DM	down-regulated	CZE followed by Orbitrap (ETD)	*Alkhalaf A et al. PLoS One. 2010;5(10):e13421*
608	Serum albumin [25-45]	P02768	Transport	Type 2 DM	up-regulated	CZE followed by Orbitrap (ETD)	*Alkhalaf A et al. PLoS One. 2010;5(10):e13421*
636	Serum albumin [25-46]	P02768	Transport	Type 2 DM	up-regulated	CZE followed by Orbitrap (ETD)	*Alkhalaf A et al. PLoS One. 2010;5(10):e13421*
661	Serum albumin [25-47]	P02768	Transport	Type 2 DM	up-regulated	CZE followed by Orbitrap (ETD)	*Alkhalaf A et al. PLoS One. 2010;5(10):e13421*
671	Collagen alpha-1 (III) chain [796-809]	P02461	Structural Component	Type 2 DM	down-regulated	CZE followed by Ion trap	*Alkhalaf A et al. PLoS One. 2010;5(10):e13421*
720	Collagen alpha-1 (III) chain [642-657]	P02461	Structural Component	Type 2 DM	down-regulated	CZE followed by Orbitrap (ETD)	*Alkhalaf A et al. PLoS One. 2010;5(10):e13421*
733	Collagen alpha-1 (I) chain [1011-1041]	P02452	Structural Component	Type 2 DM	down-regulated	CZE followed by Orbitrap (ETD)	*Alkhalaf A et al. PLoS One. 2010;5(10):e13421*
735	Uromodulin [594-606]	P07911	Defense response	Type 2 DM	down-regulated	CZE followed by Orbitrap (ETD)	*Alkhalaf A et al. PLoS One. 2010;5(10):e13421*
797	Alpha-1-antitrypsin [398-418]	P01009	Defense response	Type 2 DM	up-regulated	CZE followed by Ion trap	*Alkhalaf A et al. PLoS One. 2010;5(10):e13421*
803	oxidated Alpha-1-antitrypsin [398-418]	P01009	Defense response	Type 2 DM	up-regulated	CZE followed by Orbitrap (ETD)	*Alkhalaf A et al. PLoS One. 2010;5(10):e13421*
803	Collagen alpha-1 (I) chain [273-299]	P02452	Structural Component	Type 2 DM	down-regulated	CZE followed by Orbitrap (ETD)	*Alkhalaf A et al. PLoS One. 2010;5(10):e13421*
805	Membrane-associated progesterone receptor component 1 [53-67]	O00264	Metabolism	Type 2 DM	down-regulated	CZE followed by Orbitrap (ETD)	*Alkhalaf A et al. PLoS One. 2010;5(10):e13421*
809	Collagen alpha-1 (I) chain [273-299]	P02452	Structural Component	Type 2 DM	down-regulated	CZE followed by Orbitrap (ETD)	*Alkhalaf A et al. PLoS One. 2010;5(10):e13421*
811	Collagen alpha-1 (I) chain [819-846]	P02452	Structural Component	Type 2 DM	down-regulated	CZE followed by Orbitrap (ETD)	*Alkhalaf A et al. PLoS One. 2010;5(10):e13421*
817	Collagen alpha-1 (I) chain [588-624]	P02452	Structural Component	Type 2 DM	down-regulated	CZE followed by Orbitrap (ETD)	*Alkhalaf A et al. PLoS One. 2010;5(10):e13421*
840	Alpha-1-antitrypsin [397-418]	P01009	Defense response	Type 2 DM	up-regulated	CZE followed by Orbitrap (ETD)	*Alkhalaf A et al. PLoS One. 2010;5(10):e13421*

897	Collagen alpha-1 (I) chain [612-641]	P02452	Structural Component	Type 2 DM	down-regulated	CZE followed by Orbitrap (ETD)	*Alkhalaf A et al. PLoS One. 2010;5(10):e13421*
910	Unattributed	Unattributed	Unknown	Type 2 DM	up-regulated	CZE coupled with ESI mass spectrometry	*Mischak H. et al. Clin Sci. 2004;107:485-95*
915	Collagen alpha-1 (III) chain [610-639]	P02461	Structural Component	Type 2 DM	down-regulated	CZE followed by Orbitrap (ETD)	*Alkhalaf A et al. PLoS One. 2010;5(10):e13421*
937	Unattributed	Unattributed	Unknown	Type 2 DM with renal disease	up-regulated	CZE coupled with ESI mass spectrometry	*Mischak H. et al. Clin Sci. 2004;107:485-95*
969	Collagen alpha-2 (I) chain [844-865]	P08123	Structural Component	Type 2 DM	down-regulated	CZE followed by Ion trap	*Alkhalaf A et al. PLoS One. 2010;5(10):e13421*
973	Alpha-1-antitrypsin [378-394]	P01009	Defense response	Type 2 DM	up-regulated	CZE followed by Orbitrap (ETD)	*Alkhalaf A et al. PLoS One. 2010;5(10):e13421*
981	Unattributed	Unattributed	Unknown	Type 2 DM with renal disease	down-regulated	CZE coupled with ESI mass spectrometry	*Mischak H. et al. Clin Sci. 2004;107:485-95*
986	Transthyretin [130-147]	P02766	Transport	Type 2 DM	up-regulated	CZE followed by Ion trap	*Alkhalaf A et al. PLoS One. 2010;5(10):e13421*
995	Unattributed	Unattributed	Unknown	Type 2 DM	up-regulated	CZE coupled with ESI mass spectrometry	*Mischak H. et al. Clin Sci. 2004;107:485-95*
1,008	Collagen alpha-1 (III) chain [176 -198]	P02461	Structural Component	Type 2 DM	down-regulated	CZE followed by Orbitrap (ETD)	*Alkhalaf A et al. PLoS One. 2010;5(10):e13421*
1,009	Collagen alpha-1(I) chain	P02452	Structural Component	Type 2 DM	down-regulated with respect to Type 1 DM	CZE-MS	*Maahs DM. et al. PLoS One. 2010;5(9): e13051.*
1,011	Unattributed	Unattributed	Unknown	Type 2 DM	down-regulated	CZE coupled with ESI mass spectrometry	*Mischak H. et al. Clin Sci. 2004;107:485-95*
1,029	Collagen alpha-1 (I) chain [433-455]	P02452	Structural Component	Type 2 DM	down-regulated	CZE followed by Orbitrap (ETD)	*Alkhalaf A et al. PLoS One. 2010;5(10):e13421*
1,032	Collagen alpha-2 (I) chain [831-865]	P08123	Structural Component	Type 2 DM	down-regulated	CZE followed by Orbitrap (ETD)	*Alkhalaf A et al. PLoS One. 2010;5(10):e13421*
1,032	Collagen alpha-1 (I) chain [819-854]	P02452	Structural Component	Type 2 DM	down-regulated	CZE followed by Orbitrap (ETD)	*Alkhalaf A et al. PLoS One. 2010;5(10):e13421*
1,037	Collagen alpha-1 (I) chain[819-854]	P02452	Structural Component	Type 2 DM	down-regulated	CZE followed by Orbitrap (ETD)	*Alkhalaf A et al. PLoS One. 2010;5(10):e13421*
1,048	Unattributed	Unattributed	Unknown	Type 2 DM	down-regulated	CZE coupled with ESI mass spectrometry	*Mischak H. et al. Clin Sci. 2004;107:485-95*
1,060	Collagen alpha-1 (III) chain [886-909]	P02461	Structural Component	Type 2 DM	down-regulated	CZE followed by Orbitrap (ETD)	*Alkhalaf A et al. PLoS One. 2010;5(10):e13421*
1,078	Unattributed	Unattributed	Unknown	Type 2 DM-NP	down-regulated	CZE coupled with ESI mass spectrometry	*Rossing K. et al. J Am Soc Nephrol. 2008;19:1283–1290.*
1,094	Collagen alpha-1 (I) chain [432-455]	P02452	Structural Component	Type 2 DM	down-regulated	CZE followed by Orbitrap (ETD)	*Alkhalaf A et al. PLoS One. 2010;5(10):e13421*
1,107	Unattributed	Unattributed	Unknown	Type 2 DM	down-regulated	CZE coupled with ESI mass spectrometry	*Mischak H. et al. Clin Sci. 2004;107:485-95*

1,109	Alpha-1-antitrypsin [124-143]	P01009	Defense response	Type 2 DM	up-regulated	CZE followed by Orbitrap (ETD)	*Alkhalaf A et al. PLoS One. 2010;5(10):e13421*
1,109	Collagen alpha-1 (I) chain [769-794]	P02452	Structural Component	Type 2 DM	down-regulated	CZE followed by Orbitrap (ETD)	*Alkhalaf A et al. PLoS One. 2010;5(10):e13421*
1,128	Unattributed	Unattributed	Unknown	Type 2 DM-NP	down-regulated	CZE coupled with ESI mass spectrometry	*Rossing K. et al. J Am Soc Nephrol. 2008;19:1283–1286*
1,135	Collagen α-2 (IV) chain	J04217.1	Structural Component	Type 2 DM	down-regulated with respect to Type 1 DM	CZE-MS	*Maahs DM. et al. PLoS One. 2010;5(9): e13051.*
1,142	Unattributed	Unattributed	Unknown	Type 2 DM-NP	down-regulated	CZE coupled with ESI mass spectrometry	*Rossing K. et al. J Am Soc Nephrol. 2008;19:1283–1286*
1,142	Collagen alpha-1(I) chain	P02452	Structural Component	Type 2 DM	down-regulated with respect to Type 1 DM	CZE-MS	*Maahs DM. et al. PLoS One. 2010;5(9): e13051.*
1,160	Uromodulin	P07911	Defense response	Type 2 DM	up-regulated with respect to Type 1 DM	CZE-MS	*Maahs DM. et al. PLoS One. 2010;5(9): e13051.*
1,210	Unattributed	Unattributed	Unknown	Type 2 DM	down-regulated	CZE coupled with ESI mass spectrometry	*Mischak H. et al. Clin Sci. 2004;107:485-130*
1,219	ollagen α-5 (IV) chain precurs	P29400	Structural Component	Type 2 DM-NP and NP	up-regulated	MALDI/TOF/TOF	*Lapolla A. et al . J Mass Spectrom. 2009;44: 419-427*
1,225	Unattributed	Unattributed	Unknown	Type 2 DM	down-regulated	CZE coupled with ESI mass spectrometry	*Mischak H. et al. Clin Sci. 2004;107:485-95*
1,233	Unattributed	Unattributed	Unknown	Type 2 DM with renal disease	down-regulated	CZE coupled with ESI mass spectrometry	*Mischak H. et al. Clin Sci. 2004;107:485-95*
1,237	Collagen alpha-1 (I) chain [766-794]	P02452	Structural Component	Type 2 DM	down-regulated	CZE followed by Ion trap	*Alkhalaf A et al. PLoS One. 2010;5(10):e13421*
1,245	Collagen alpha-1 (I) chain [818-846]	P02452	Structural Component	Type 2 DM	down-regulated	CZE followed by Orbitrap (ETD)	*Alkhalaf A et al. PLoS One. 2010;5(10):e13421*
1,325	Collagen α-1 (III) chain	P02461	Structural Component	Type 2 DM-NP	up-regulated	CZE coupled with ESI mass spectrometry	*Rossing K. et al. J Am Soc Nephrol. 2008;19:1283–1286*
1,327	Collagen type 3 α1	Unknown	Structural Component	Type 2 DM-NP	down-regulated	CZE coupled with ESI mass spectrometry	*Rossing K. et al. J Am Soc Nephrol. 2008;19:1283–1286*
1,327	Collagen alpha-1 (III) chain	P02461	Structural Component	Type 2 DM	down-regulated with respect to Type 1 DM	CZE-MS	*Maahs DM. et al. PLoS One. 2010;5(9): e13051.*
1,332	Unattributed	Unattributed	Unknown	Type 2 DM	down-regulated	CZE coupled with ESI mass spectrometry	*Mischak H. et al. Clin Sci. 2004;107:485-95*
1,333	Unattributed	Unattributed	Unknown	Type 2 DM	up-regulated	CZE coupled with ESI mass spectrometry	*Mischak H. et al. Clin Sci. 2004;107:485-95*

1,354	Unattributed	Unattributed	Unknown	Type 2 DM-NP	down-regulated	CZE coupled with ESI mass spectrometry	*Rossing K. et al. J Am Soc Nephrol. 2008;19:1283–1286*
1,358	Collagen alpha-1(I) chain	P02452	Structural Component	Type 2 DM	down-regulated with respect to Type 1 DM	CZE-MS	*Maahs DM. et al. PLoS One. 2010;5(9): e13051.*
1,368	Unattributed	Unattributed	Unknown	Type 2 DM with renal disease	down-regulated	CZE coupled with ESI mass spectrometry	*Mischak H. et al. Clin Sci. 2004;107:485-95*
1,397	Unattributed	Unattributed	Unknown	Type 2 DM-NP	down-regulated	CZE coupled with ESI mass spectrometry	*Rossing K. et al. J Am Soc Nephrol. 2008;19:1283–1290,*
1,404	Unattributed	Unattributed	Unknown	Type 2 DM	up-regulated	CZE coupled with ESI mass spectrometry	*Mischak H. et al. Clin Sci. 2004;107:485-95*
1,408	Unattributed	Unattributed	Unknown	Type 2 DM with renal disease	down-regulated	CZE coupled with ESI mass spectrometry	*Mischak H. et al. Clin Sci. 2004;107:485-95*
1,425	Unattributed	Unattributed	Unknown	Type 2 DM with renal disease	down-regulated	CZE coupled with ESI mass spectrometry	*Mischak H. et al. Clin Sci. 2004;107:485-95*
1,439	Collagen alpha-1 (III) chain	P02461	Structural Component	Type 2 DM	down-regulated with respect to Type 1 DM	CZE-MS	*Maahs DM. et al. PLoS One. 2010;5(9): e13051.*
1,443	Unattributed	Unattributed	Unknown	Type 2 DM	up-regulated	CZE coupled with ESI mass spectrometry	*Mischak H. et al. Clin Sci. 2004;107:485-95*
1,452	Collagen alpha-1 (III) chain	P02461	Structural Component	Type 2 DM	down-regulated with respect to Type 1 DM	CZE-MS	*Maahs DM. et al. PLoS One. 2010;5(9): e13051.*
1,463	Unattributed	Unattributed	Unknown	Type 2 DM with renal disease	down-regulated	CZE coupled with ESI mass spectrometry	*Mischak H. et al. Clin Sci. 2004;107:485-95*
1,466	Unattributed	Unattributed	Unknown	Type 2 DM with renal disease	up-regulated	CZE coupled with ESI mass spectrometry	*Mischak H. et al. Clin Sci. 2004;107:485-95*
1,484	Unattributed	Unattributed	Unknown	Type 2 DM with renal disease	up-regulated	CZE coupled with ESI mass spectrometry	*Mischak H. et al. Clin Sci. 2004;107:485-95*
1,485	Unattributed	Unattributed	Unknown	Type 2 DM	down-regulated	CZE coupled with ESI mass spectrometry	*Mischak H. et al. Clin Sci. 2004;107:485-95*
1,486	Collagen α-1 (I) chain [829 to 835]	P02452	Structural Component	Type 2 DM-NP	down-regulated	CZE coupled with ESI mass spectrometry	*Rossing K. et al. J Am Soc Nephrol. 2008;19:1283–1286*
1,487	Unattributed	Unattributed	Unknown	Type 2 DM-NP	down-regulated	CZE coupled with ESI mass spectrometry	*Rossing K. et al. J Am Soc Nephrol. 2008;19:1283–1286*
1,524	Unattributed	Unattributed	Unknown	Type 2 DM with renal disease	down-regulated	CZE coupled with ESI mass spectrometry	*Mischak H. et al. Clin Sci. 2004;107:485-95*
1,527	Unattributed	Unattributed	Unknown	Type 2 DM-NP	down-regulated	CZE coupled with ESI mass spectrometry	*Rossing K. et al. J Am Soc Nephrol. 2008;19:1283–1286*

1,530	Unattributed	Unattributed	Unknown	Type 2 DM with renal disease	down-regulated	CZE coupled with ESI mass spectrometry	*Mischak H. et al. Clin Sci. 2004;107:485-95*
1,540	Unattributed	Unattributed	Unknown	Type 2 DM with renal disease	down-regulated	CZE coupled with ESI mass spectrometry	*Mischak H. et al. Clin Sci. 2004;107:485-95*
1,546	Unattributed	Unattributed	Unknown	Type 2 DM with renal disease	down-regulated	CZE coupled with ESI mass spectrometry	*Mischak H. et al. Clin Sci. 2004;107:485-95*
1,550	Unattributed	Unattributed	Unknown	Type 2 DM-NP	down-regulated	CZE coupled with ESI mass spectrometry	*Rossing K. et al. J Am Soc Nephrol. 2008;19:1283–1286*
1,562	Unattributed	Unattributed	Unknown	Type 2 DM with renal disease	down-regulated	CZE coupled with ESI mass spectrometry	*Mischak H. et al. Clin Sci. 2004;107:485-95*
1,567	Unattributed	Unattributed	Unknown	Type 2 DM with renal disease	up-regulated	CZE coupled with ESI mass spectrometry	*Mischak H. et al. Clin Sci. 2004;107:485-95*
1,578	Unattributed	Unattributed	Unknown	Type 2 DM with renal disease	down-regulated	CZE coupled with ESI mass spectrometry	*Mischak H. et al. Clin Sci. 2004;107:485-95*
1,595	Unattributed	Unattributed	Unknown	Type 2 DM with renal disease	down-regulated	CZE coupled with ESI mass spectrometry	*Mischak H. et al. Clin Sci. 2004;107:485-95*
1,597	Unattributed	Unattributed	Unknown	Type 2 DM with renal disease	up-regulated	CZE coupled with ESI mass spectrometry	*Mischak H. et al. Clin Sci. 2004;107:485-95*
1,610	Unattributed	Unattributed	Unknown	Type 2 DM	down-regulated	CZE coupled with ESI mass spectrometry	*Mischak H. et al. Clin Sci. 2004;107:485-95*
1,652	Unattributed	Unattributed	Unknown	Type 2 DM with renal disease	down-regulated	CZE coupled with ESI mass spectrometry	*Mischak H. et al. Clin Sci. 2004;107:485-95*
1,680	Unattributed	Unattributed	Unknown	Type 2 DM with renal disease	down-regulated	CZE coupled with ESI mass spectrometry	*Mischak H. et al. Clin Sci. 2004;107:485-95*
1,680	Uromodulin	P07911	Defense response	Type 2 DM	up-regulated with respect to Type 1 DM	CZE-MS	*Maahs DM. et al. PLoS One. 2010;5(9): e13051.*
1,699	Unattributed	Unattributed	Unknown	Type 2 DM-NP	down-regulated	CZE coupled with ESI mass spectrometry	*Rossing K. et al. J Am Soc Nephrol. 2008;19:1283–1286*
1,717	Unattributed	Unattributed	Unknown	Type 2 DM-NP	down-regulated	CZE coupled with ESI mass spectrometry	*Rossing K. et al. J Am Soc Nephrol. 2008;19:1283–1286*
1,717	Unattributed	Unattributed	Unknown	Type 2 DM	down-regulated	CZE coupled with ESI mass spectrometry	*Mischak H. et al. Clin Sci. 2004;107:485-95*
1,726	Unattributed	Unattributed	Unknown	Type 2 DM-NP	down-regulated	CZE coupled with ESI mass spectrometry	*Rossing K. et al. J Am Soc Nephrol. 2008;19:1283–1286*
1,733	Unattributed	Unattributed	Unknown	Type 2 DM-NP	down-regulated	CZE coupled with ESI mass spectrometry	*Rossing K. et al. J Am Soc Nephrol. 2008;19:1283–1286*
1,789	Unattributed	Unattributed	Unknown	Type 2 DM	up-regulated	CZE coupled with ESI mass spectrometry	*Mischak H. et al. Clin Sci. 2004;107:485-95*

1,818	Unattributed	Unattributed	Unknown	Type 2 DM	down-regulated	CZE coupled with ESI mass spectrometry	*Mischak H. et al. Clin Sci. 2004;107:485-95*
1,819	Unattributed	Unattributed	Unknown	Type 2 DM	up-regulated	CZE coupled with ESI mass spectrometry	*Mischak H. et al. Clin Sci. 2004;107:485-95*
1,844	Unattributed	Unattributed	Unknown	Type 2 DM-NP	down-regulated	CZE coupled with ESI mass spectrometry	*Rossing K. et al. J Am Soc Nephrol. 2008;19:1283–1286*
1,847	Collagen alpha-1(I) chain	P02452	Structural Component	Type 2 DM	down-regulated with respect to Type 1 DM	CZE-MS	*Maahs DM. et al. PLoS One. 2010;5(9): e13051.*
1,851	Unattributed	Unattributed	Unknown	Type 2 DM with renal disease	up-regulated	CZE coupled with ESI mass spectrometry	*Mischak H. et al. Clin Sci. 2004;107:485-95*
1,864	Unattributed	Unattributed	Unknown	Type 2 DM with renal disease	down-regulated	CZE coupled with ESI mass spectrometry	*Mischak H. et al. Clin Sci. 2004;107:485-95*
1,866	Collagen α-1 (I) chain [279 to 299]	P02452	Structural Component	Type 2 DM-NP	down-regulated	CZE coupled with ESI mass spectrometry	*Rossing K. et al. J Am Soc Nephrol. 2008;19:1283–1286*
1,867	Unattributed	Unattributed	Unknown	Type 2 DM with renal disease	up-regulated	CZE coupled with ESI mass spectrometry	*Mischak H. et al. Clin Sci. 2004;107:485-95*
1,873	Unattributed	Unattributed	Unknown	Type 2 DM	up-regulated	CZE coupled with ESI mass spectrometry	*Mischak H. et al. Clin Sci. 2004;107:485-95*
1,886	Unattributed	Unattributed	Unknown	Type 2 DM-NP	down-regulated	CZE coupled with ESI mass spectrometry	*Rossing K. et al. J Am Soc Nephrol. 2008;19:1283–1286*
1,893	Unattributed	Unattributed	Unknown	Type 2 DM-NP	down-regulated	CZE coupled with ESI mass spectrometry	*Rossing K. et al. J Am Soc Nephrol. 2008;19:1283–1286*
1,912	uromodulin precursor	P07911	Defense response	Type 2 DM-NP and NP	down-regulated	MALDI/TOF/TOF	*Lapolla A. et al. J Mass Spectrom. 2009;44: 419-426*
1,937	Collagen α-2 (I) chain [844 to 865]	P08123	Structural Component	Type 2 DM-NP	down-regulated	CZE coupled with ESI mass spectrometry	*Rossing K. et al. J Am Soc Nephrol. 2008;19:1283–1286*
1,955	Unattributed	Unattributed	Unknown	Type 2 DM	up-regulated	CZE coupled with ESI mass spectrometry	*Mischak H. et al. Clin Sci. 2004;107:485-95*
1,971	Unattributed	Unattributed	Unknown	Type 2 DM	up-regulated	CZE coupled with ESI mass spectrometry	*Mischak H. et al. Clin Sci. 2004;107:485-95*
2,020	Collagen alpha-1 (III) chain	P02461	Structural Component	Type 2 DM	down-regulated with respect to Type 1 DM	CZE-MS	*Maahs DM. et al. PLoS One. 2010;5(9): e13051.*
2,032	Unattributed	Unattributed	Unknown	Type 2 DM	up-regulated	CZE coupled with ESI mass spectrometry	*Mischak H. et al. Clin Sci. 2004;107:485-95*
2,039	Uromodulin [589 to 607]	P07911	Defense response	Type 2 DM-NP	down-regulated	CZE coupled with ESI mass spectrometry	*Rossing K. et al. J Am Soc Nephrol. 2008;19:1283–1286*

2,047	Unattributed	Unattributed	Unknown	Type 2 DM-NP	down-regulated	CZE coupled with ESI mass spectrometry	*Rossing K. et al. J Am Soc Nephrol. 2008;19:1283–1286*
2,049	Collagen α-1 (I) chain precursor	P02452	Structural Component	Type 2 DM-NP and NP	up-regulated	MALDI/TOF/TOF	*Lapolla A. et al. J Mass Spectrom. 2009;44: 419-425*
2,061	Unattributed	Unattributed	Unknown	Type 2 DM	up-regulated	CZE coupled with ESI mass spectrometry	*Mischak H. et al. Clin Sci. 2004;107:485-95*
2,063	Collagen alpha-1 (III) chain	P02461	Structural Component	Type 2 DM	down-regulated with respect to Type 1 DM	CZE-MS	*Maahs DM. et al. PLoS One. 2010;5(9): e13051.*
2,092	Unattributed	Unattributed	Unknown	Type 2 DM	up-regulated	CZE coupled with ESI mass spectrometry	*Mischak H. et al. Clin Sci. 2004;107:485-95*
2,119	Unattributed	Unattributed	Unknown	Type 2 DM	down-regulated	CZE coupled with ESI mass spectrometry	*Mischak H. et al. Clin Sci. 2004;107:485-95*
2,186	Unattributed	Unattributed	Unknown	Type 2 DM	up-regulated	CZE coupled with ESI mass spectrometry	*Mischak H. et al. Clin Sci. 2004;107:485-95*
2,192	Collagen α-1 (I) chain	P02452	Structural Component	Type 2 DM-NP	down-regulated	CZE coupled with ESI mass spectrometry	*Rossing K. et al. J Am Soc Nephrol. 2008;19:1283–1286*
2,192	Collagen alpha-1(I) chain	P02452	Structural Component	Type 2 DM	down-regulated with respect to Type 1 DM	CZE-MS	*Maahs DM. et al. PLoS One. 2010;5(9): e13051.*
2,228	Unattributed	Unattributed	Unknown	Type 2 DM with renal disease	up-regulated	CZE coupled with ESI mass spectrometry	*Mischak H. et al. Clin Sci. 2004;107:485-95*
2,230	Unattributed	Unattributed	Unknown	Type 2 DM	up-regulated	CZE coupled with ESI mass spectrometry	*Mischak H. et al. Clin Sci. 2004;107:485-95*
2,257	Unattributed	Unattributed	Unknown	Type 2 DM	down-regulated	CZE coupled with ESI mass spectrometry	*Mischak H. et al. Clin Sci. 2004;107:485-95*
2,339	Unattributed	Unattributed	Unknown	Type 2 DM-NP	down-regulated	CZE coupled with ESI mass spectrometry	*Rossing K. et al. J Am Soc Nephrol. 2008;19:1283–1286*
2,339	Collagen alpha-1(I) chain	P02452	Structural Component	Type 2 DM	down-regulated with respect to Type 1 DM	CZE-MS	*Maahs DM. et al. PLoS One. 2010;5(9): e13051.*
2,377	Collagen α-1 (I) chain [227 to 250]	P02452	Structural Component	Type 2 DM-NP	down-regulated	CZE coupled with ESI mass spectrometry	*Rossing K. et al. J Am Soc Nephrol. 2008;19:1283–1286*
2,406	Unattributed	Unattributed	Unknown	Type 2 DM with renal disease	up-regulated	CZE coupled with ESI mass spectrometry	*Mischak H. et al. Clin Sci. 2004;107:485-95*
2,424	Unattributed	Unattributed	Unknown	Type 2 DM	down-regulated	CZE coupled with ESI mass spectrometry	*Mischak H. et al. Clin Sci. 2004;107:485-95*
2,427	Unattributed	Unattributed	Unknown	Type 2 DM with renal disease	up-regulated	CZE coupled with ESI mass spectrometry	*Mischak H. et al. Clin Sci. 2004;107:485-95*

2,430	Collagen alpha-1(I) chain	P02452	Structural Component	Type 2 DM	down-regulated with respect to Type 1 DM	CZE-MS	*Maahs DM. et al. PLoS One. 2010;5(9): e13051.*
2,465	Unattributed	Unattributed	Unknown	Type 2 DM with renal disease	up-regulated	CZE coupled with ESI mass spectrometry	*Mischak H. et al. Clin Sci. 2004;107:485-95*
2,487	Collagen alpha-1(I) chain	P02452	Structural Component	Type 2 DM	down-regulated with respect to Type 1 DM	CZE-MS	*Maahs DM. et al. PLoS One. 2010;5(9): e13051.*
2,494	Unattributed	Unattributed	Unknown	Type 2 DM with renal disease	up-regulated	CZE coupled with ESI mass spectrometry	*Mischak H. et al. Clin Sci. 2004;107:485-95*
2,522	Unattributed	Unattributed	Unknown	Type 2 DM with renal disease	up-regulated	CZE coupled with ESI mass spectrometry	*Mischak H. et al. Clin Sci. 2004;107:485-95*
2,541	Unattributed	Unattributed	Unknown	Type 2 DM with renal disease	up-regulated	CZE coupled with ESI mass spectrometry	*Mischak H. et al. Clin Sci. 2004;107:485-95*
2,551	Unattributed	Unattributed	Unknown	Type 2 DM-NP	down-regulated	CZE coupled with ESI mass spectrometry	*Rossing K. et al. J Am Soc Nephrol. 2008;19:1283–1286*
2,574	Unattributed	Unattributed	Unknown	Type 2 DM-NP	down-regulated	CZE coupled with ESI mass spectrometry	*Rossing K. et al. J Am Soc Nephrol. 2008;19:1283–1286*
2,587	Unattributed	Unattributed	Unknown	Type 2 DM	down-regulated	CZE coupled with ESI mass spectrometry	*Mischak H. et al. Clin Sci. 2004;107:485-95*
2,644	Unattributed	Unattributed	Unknown	Type 2 DM	down-regulated	CZE coupled with ESI mass spectrometry	*Mischak H. et al. Clin Sci. 2004;107:485-95*
2,670	Unattributed	Unattributed	Unknown	Type 2 DM	up-regulated	CZE coupled with ESI mass spectrometry	*Mischak H. et al. Clin Sci. 2004;107:485-95*
2,687	Collagen alpha-1(I) chain	P02452	Structural Component	Type 2 DM	down-regulated with respect to Type 1 DM	CZE-MS	*Maahs DM. et al. PLoS One. 2010;5(9): e13051.*
2,698	Unattributed	Unattributed	Unknown	Type 2 DM	down-regulated	CZE coupled with ESI mass spectrometry	*Mischak H. et al. Clin Sci. 2004;107:485-95*
2,711	Unattributed	Unattributed	Unknown	Type 2 DM with renal disease	down-regulated	CZE coupled with ESI mass spectrometry	*Mischak H. et al. Clin Sci. 2004;107:485-95*
2,734	Unattributed	Unattributed	Unknown	Type 2 DM-NP	down-regulated	CZE coupled with ESI mass spectrometry	*Rossing K. et al. J Am Soc Nephrol. 2008;19:1283–1286*
2,749	Unattributed	Unattributed	Unknown	Type 2 DM-NP	down-regulated	CZE coupled with ESI mass spectrometry	*Rossing K. et al. J Am Soc Nephrol. 2008;19:1283–1286*
2,751	Unattributed	Unattributed	Unknown	Type 2 DM-NP	down-regulated	CZE coupled with ESI mass spectrometry	*Rossing K. et al. J Am Soc Nephrol. 2008;19:1283–1286*
2,791	Unattributed	Unattributed	Unknown	Type 2 DM with renal disease	up-regulated	CZE coupled with ESI mass spectrometry	*Mischak H. et al. Clin Sci. 2004;107:485-95*

2,796	Unattributed	Unattributed	Unknown	Type 2 DM	up-regulated	CZE coupled with ESI mass spectrometry	*Mischak H. et al. Clin Sci. 2004;107:485-95*
2,803	Unattributed	Unattributed	Unknown	Type 2 DM-NP	down-regulated	CZE coupled with ESI mass spectrometry	*Rossing K. et al. J Am Soc Nephrol. 2008;19:1283–1286*
2,937	Unattributed	Unattributed	Unknown	Type 2 DM with renal disease	up-regulated	CZE coupled with ESI mass spectrometry	*Mischak H. et al. Clin Sci. 2004;107:485-95*
2,959	Unattributed	Unattributed	Unknown	Type 2 DM with renal disease	up-regulated	CZE coupled with ESI mass spectrometry	*Mischak H. et al. Clin Sci. 2004;107:485-95*
2,995	Unattributed	Unattributed	Unknown	Type 2 DM	down-regulated	CZE coupled with ESI mass spectrometry	*Mischak H. et al. Clin Sci. 2004;107:485-95*
3,081	Unattributed	Unattributed	Unknown	Type 2 DM-NP	down-regulated	CZE coupled with ESI mass spectrometry	*Rossing K. et al. J Am Soc Nephrol. 2008;19:1283–1286*
3,092	Collagen alpha-1(I) chain	P02452	Structural Component	Type 2 DM	down-regulated with respect to Type 1 DM	CZE-MS	*Maahs DM. et al. PLoS One. 2010;5(9): e13051.*
3,109	Unattributed	Unattributed	Unknown	Type 2 DM with renal disease	down-regulated	CZE coupled with ESI mass spectrometry	*Mischak H. et al. Clin Sci. 2004;107:485-95*
3,224	Unattributed	Unattributed	Unknown	Type 2 DM with renal disease	down-regulated	CZE coupled with ESI mass spectrometry	*Mischak H. et al. Clin Sci. 2004;107:485-95*
3,246	Unattributed	Unattributed	Unknown	Type 2 DM	up-regulated	CZE coupled with ESI mass spectrometry	*Mischak H. et al. Clin Sci. 2004;107:485-95*
3,293	Unattributed	Unattributed	Unknown	Type 2 DM with renal disease	down-regulated	CZE coupled with ESI mass spectrometry	*Mischak H. et al. Clin Sci. 2004;107:485-95*
3,296	Unattributed	Unattributed	Unknown	Type 2 DM	down-regulated	CZE coupled with ESI mass spectrometry	*Mischak H. et al. Clin Sci. 2004;107:485-95*
3,334	Unattributed	Unattributed	Unknown	Type 2 DM-NP	up-regulated	CZE coupled with ESI mass spectrometry	*Rossing K. et al. J Am Soc Nephrol. 2008;19:1283–1290.*
3,364	Unattributed	Unattributed	Unknown	Type 2 DM-NP	up-regulated	CZE coupled with ESI mass spectrometry	*Rossing K. et al. J Am Soc Nephrol. 2008;19:1283–1290.*
3,479	Unattributed	Unattributed	Unknown	Type 2 DM with renal disease	down-regulated	CZE coupled with ESI mass spectrometry	*Mischak H. et al. Clin Sci. 2004;107:485-95*
3,617	Collagen alpha-2(I) chain	P08123	Structural Component	Type 2 DM	down-regulated with respect to Type 1 DM	CZE-MS	*Maahs DM. et al. PLoS One. 2010;5(9): e13051.*
3,658	Unattributed	Unattributed	Unknown	Type 2 DM-NP	down-regulated	CZE coupled with ESI mass spectrometry	*Rossing K. et al. J Am Soc Nephrol. 2008;19:1283–1286*
3,670	Unattributed	Unattributed	Unknown	Type 2 DM-NP	up-regulated	CZE coupled with ESI mass spectrometry	*Rossing K. et al. J Am Soc Nephrol. 2008;19:1283–1286*

3,802	Collagen alpha-2(I) chain	P08123	Structural Component	Type 2 DM	down-regulated with respect to Type 1 DM	CZE-MS	*Maahs DM. et al. PLoS One. 2010;5(9): e13051.*
4,714	Unattributed	Unattributed	Unknown	Type 2 DM with renal disease	up-regulated	CZE coupled with ESI mass spectrometry	*Mischak H. et al. Clin Sci. 2004;107:485-95*
4,864	Unattributed	Unattributed	Unknown	Type 2 DM	down-regulated	CZE coupled with ESI mass spectrometry	*Mischak H. et al. Clin Sci. 2004;107:485-95*
4,962	Unattributed	Unattributed	Unknown	Type 2 DM	up-regulated	CZE coupled with ESI mass spectrometry	*Mischak H. et al. Clin Sci. 2004;107:485-95*
5,574	Unattributed	Unattributed	Unknown	Type 2 DM-NP	up-regulated	CZE coupled with ESI mass spectrometry	*Rossing K. et al. J Am Soc Nephrol. 2008;19:1283–1286*
9,181	Unattributed	Unattributed	Unknown	Type 2 DM with renal disease	up-regulated	CZE coupled with ESI mass spectrometry	*Mischak H. et al. Clin Sci. 2004;107:485-136*
10,046	Unattributed	Unattributed	Unknown	Type 2 DM with renal disease	up-regulated	CZE coupled with ESI mass spectrometry	*Mischak H. et al. Clin Sci. 2004;107:485-135*
11,773	Ig Kappa Chain C region	P01834	Defense response	Type 2 DM-NP	upregulated	DIGE followed MALDI-TOF-MS	*Jiang H. et al. Diabetes Metab Res Rev 2009; 25: 232–241.*
11,774	β_2-microglobulin	P61769	Defense response	Type 2 DM-NP with Proteinuria	up-regulated	anion exchange, reversed-phase fractionation, gel electrophoresis and SELDI-TOF MS	*Dihazi H. et al. Clin Chem. 2007;53(9):1636-45*
13,234	Calgranulin B	P06702	Defense response	Type 2 DM-NP	up-regulated	Using DIGE followed LC/MS/MS peptide analysis	*Rao VP et al. Diabetes care. 2007;30:629-37*
14,766	UbA52	P62987	Metabolism	Type 2 DM-NP with Proteinuria	up-regulated	anion exchange, reversed-phase fractionation, gel electrophoresis and SELDI-TOF MS	*Dihazi H. et al. Clin Chem. 2007;53(9):1636-45*
15,877	Transthyretin precursor	P02766	Metabolism	Type 2 DM-NP	down-regulated	Using DIGE followed LC/MS/MS peptide analysis	*Rao VP et al. Diabetes care. 2007;30:629-37*
15,991	Transthyretin	P02766	Transport	Type 2 DM	down-regulated	Two-Dimensional Liquid Chromatography followed by MALDI	*Riaz S. et al. Diabetes Technol Ther. 2010;12(12):979-88*
15,991	Transthyretin precursor	P02766	Transport	Type 2 DM-NP	downregulated	DIGE followed MALDI-TOF-MS	*Jiang H. et al. Diabetes Metab Res Rev 2009; 25: 232–241.*
21,243	Prostaglandin-H2D-isomerase precursor	P41222	Metabolism	Type 2 DM-NP	upregulated	DIGE followed MALDI-TOF-MS	*Jiang H. et al. Diabetes Metab Res Rev 2009; 25: 232–241.*

22,995	Plasma retinol-binding protein	P02753	Signal trasduction	Type 2 DM-NP	down-regulated	DIGE followed LC/MS/MS peptide analysis	*Rao VP et al. Diabetes care. 2007;30:629-37*
23,371	Retinol binding protein 4	P02753	Transport	Type 2 DM	up-regulated	Two-Dimensional Liquid Chromatography followed by MALDI	*Riaz S. et al. Diabetes Technol Ther. 2010;12(12):979-88*
23,371	Plasma retinol-binding protei precursor	P02753	Signal trasduction	Type 2 DM-NP	upregulated	DIGE followed MALDI-TOF-MS	*Jiang H. et al. Diabetes Metab Res Rev 2009; 25: 232–241.*
23,725	α1-acid glycoprotein precursor	P02763	Transport	Type 2 DM-NP	upregulated	DIGE followed MALDI-TOF-MS	*Jiang H. et al. Diabetes Metab Res Rev 2009; 25: 232–241.*
30,759	Apolipoprotein A-I	P02647	Metabolism	Type 2 DM-NP	down-regulated	Using DIGE followed LC/MS/MS peptide analysis	*Rao VP et al. Diabetes care. 2007;30:629-39*
34,079	Zinc- α_2-glycoprotein	P25311	Metabolism	Type 2 DM	up-regulated	Two-Dimensional Liquid Chromatography followed by MALDI	*Riaz S. et al. Diabetes Technol Ther. 2010;12(12):979-88*
34,079	Zinc-α2-glycoprotein precursor	P25311	Metabolism	Type 2 DM-NP	upregulated	DIGE followed MALDI-TOF-MS	*Jiang H. et al. Diabetes Metab Res Rev 2009; 25: 232–241.*
34,223	Zinc-α_2-glycoprotein	P25311	Defense response	Type 2 DM-NP	up-regulated	DIGE followed LC/MS/MS peptide analysis	*Rao VP et al. Diabetes care. 2007;30:629-37*
39,300	α_2-HS-glycoprotein	P02765	Metabolism	Type 2 DM-NP	up-regulated	DIGE followed LC/MS/MS peptide analysis	*Rao VP et al. Diabetes care. 2007;30:629-37*
39,886	α1-microglobulin/bikunin precursor (AMBP)	P02760	Defense response	Type 2 DM	down-regulated	Two-Dimensional Liquid Chromatography followed by MALDI	*Riaz S. et al. Diabetes Technol Ther. 2010;12(12):979-88*
39,886	α1-microglobulin/bikunin precursor (AMBP)	P02760	Defense response	Type 2 DM-NP	downregulated	DIGE followed MALDI-TOF-MS	*Jiang H. et al. Diabetes Metab Res Rev 2009; 25: 232–241.*
45,860	Haptoglobin precursor	P00738	Metabolism	Type 2 DM	down-regulated	Two-Dimensional Liquid Chromatography followed by MALDI	*Riaz S. et al. Diabetes Technol Ther. 2010;12(12):979-88*
45,861	Haptoglobin precursor	P00738	Metabolism	Type 2 DM-NP	downregulated	DIGE followed MALDI-TOF-MS	*Jiang H. et al. Diabetes Metab Res Rev 2009; 25: 232–241.*
46,707	α_1-antitrypsin	P01009	Defense response	Type 2 DM-NP	up-regulated	DIGE followed LC/MS/MS peptide analysis	*Rao VP et al. Diabetes care. 2007;30:629-37*

51,643	α_1-microglobulin/bikunin precursor	P02790	Transport	Type 2 DM-NP	down-regulated	DIGE followed LC/MS/MS peptide analysis	*Rao VP et al. Diabetes care. 2007;30:629-37*
51,643	Hemopexin	P02790	Defense response	Type 2 DM-NP	up-regulated	DIGE followed LC/MS/MS peptide analysis	*Rao VP et al. Diabetes care. 2007;30:629-37*
52,964	Vitamin D-binding protein	P02774	Transport	Type 2 DM-NP	up-regulated	DIGE followed LC/MS/MS peptide analysis	*Rao VP et al. Diabetes care. 2007;30:629-37*
54,239	α_{1B}-Glycoprotein	P04217	Function not assigned	Type 2 DM-NP	up-regulated	DIGE followed LC/MS/MS peptide analysis	*Rao VP et al. Diabetes care. 2007;30:629-37*
71,317	Albumin	P02768	Transport	Type 2 DM	up-regulated	Two-Dimensional Liquid Chromatography followed by MALDI	*Riaz S. et al. Diabetes Technol Ther. 2010;12(12):979-88*
71,317	Serum albumin precursor	P02768	Transport	Type 2 DM-NP	upregulated	DIGE followed MALDI-TOF-MS	*Jiang H. et al. Diabetes Metab Res Rev 2009; 25: 232–241.*
72,451	Uromodulin precursor	P07911	Defense response	Type 2 DM-NP	downregulated	DIGE followed MALDI-TOF-MS	*Jiang H. et al. Diabetes Metab Res Rev 2009; 25: 232–241.*
72,984	Kininogen precursor	P01042	Defense response	Type 2 DM-NP	upregulated	DIGE followed MALDI-TOF-MS	*Jiang H. et al. Diabetes Metab Res Rev 2009; 25: 232–241.*
97,853	Epithelial-cadherin	P12830	Cell adhesion	Type 2 DM	up-regulated	Two-Dimensional Liquid Chromatography followed by MALDI	*Riaz S. et al. Diabetes Technol Ther. 2010;12(12):979-88*
97,853	Epithelial-cadherin	P12830	Cell adhesion	Type 2 DM-NP	upregulated	DIGE followed MALDI-TOF-MS	*Jiang H. et al. Diabetes Metab Res Rev 2009; 25: 232–241.*
